# The cause of death in bacterial meningitis

**DOI:** 10.1186/s12879-020-4899-x

**Published:** 2020-02-27

**Authors:** A. Sharew, J. Bodilsen, B. R. Hansen, H. Nielsen, C. T. Brandt

**Affiliations:** 1 Department of pulmonary and Infectious Diseases, Nordsjællands Hospital, University of Copenhagen, Dyrehavevej 29, 3400 Hilleroed, Denmark; 2 Department of Infectious Diseases, Aalborg University Hospital, Aalborg, Denmark; 3 Department of Infectious Diseases, University Hospital Copenhagen Hvidovre, Hvidovre, Denmark; 4 Department of Clinical Medicine, Aalborg University, Aalborg, Denmark; 5 Department of Clinical Medicine, University of Copenhagen, Copenhagen, Denmark

**Keywords:** Bacterial meningitis, Cause of death, Systemic complications, Central nervous system complications, Sepsis, Brain herniation

## Abstract

**Background:**

Death from bacterial meningitis is rarely attributed to the actual event causing death.

The present study therefore categorized and characterized the cause and time of death due to bacterial meningitis.

**Methods:**

In a cohort of patients > 15 years of age with community acquired bacterial meningitis the medical records were reviewed, and a clinical cause of death categorized into six main categories: 1) CNS complications, 2) Systemic complications, 3) Combination of systemic and CNS complications, 4) Sudden death, 5) Withdrawal of care, or 6) Unknown.

**Results:**

We identified 358 patients of which 84 (23%) died in-hospital. Causes of death were ascribed to CNS complications in 43%, Systemic complications in 39%, Combined CNS and systemic complications in 4%, Sudden death in 7% and withdrawal of care in 5%. Brain herniation, circulatory failure, intractable seizures and other brain injury were the most common specific causes of death within 14 days from admission (55%).

**Conclusion:**

Fatal complications due to the primary infection – meningitis - is most common within 14 days of admission. The diversity of complications causing death in meningitis suggest that determining the clinical cause of death is essential to the evaluation of novel treatment strategies.

## Background

Despite advances in clinical care, bacterial meningitis remains a severe disease with a high risk of complications that may lead to death or severe sequelae [1]. These complications can be classified as systemic or local hence referring to shock, respiratory failure, organ failure, coagulation disorders or the intracranial complications stroke, seizures or brain herniation [2]. Yet, death from meningitis is rarely attributed to a diagnosed or suspected complication.

Previous studies have identified that systemic disease complications - septicemia and respiratory failure - was the primary cause of death among elderly patients with bacterial meningitis whereas local complications to the brain infection, i.e. brain herniation, dominates among younger patients [3, 4]. Also, it has been suggested that causes not directly related to meningitis are responsible for late deaths more than 2 weeks after admission, whereas fatal outcome before day 14 is more likely to be attributed to meningitis and complications related to meningitis [5].

A recent study in patients with septicemia identified different sepsis profiles based on individual organ failure complications [6]. These profiles were closely related to the response to fluid resuscitation and case fatality rate. Similarly, determining the cause of death in patients presenting with acute bacterial meningitis could improve the interpretation of clinical studies and the identification of risk factors. The present study therefore aimed to determine the causes and time of death due to meningitis in a population-based cohort.

## Method

### Setting and study population

We included patients diagnosed with community acquired bacterial meningitis from January 1. 1998 to 31. 12.2014. from the University Hospital in the North Jutland Region and from January 1. 2003 to 31.12.2014 from two university hospitals in the Capital Region of Denmark as described previously [7].

Patients were eligible to be included if the following inclusion criteria were fulfilled: Patients were older than 15 years of age, had a clinical presentation strongly suggesting bacterial meningitis (headache, fever, stiffness of the neck, petechiae, confusion or impaired level of consciousness) and ≥ 1 of the following inclusion criteria [7–9]:

1. Positive cerebrospinal fluid (CSF) culture
2. Positive blood culture and one or more of the following CSF findings: > 10 leukocytes (× 10^6^ cells/L); glucose index < 0.23; CSF glucose < 1.9 mmol/L; protein > 2.2 g/L
3. Presence of bacteria in Gram stain of CSF.
4. Non-culture identification of bacteria in CSF by either gene amplification or antigen test

Exclusion criteria:

1. Patients with unidentified cause of CSF inflammation
2. Nosocomial meningitis [10]
3. Patients whose records/files could not be retrieved

Patients were a priori divided into three groups based on time of death since admission – early (< 48 h), intermediate (3 to 14 days) and late death (> 14 days) [2, 5].

Time of arrival at hospital as noted by ambulance staff, nurses or secretaries in the Emergency Department was used for calculation of time to antibiotic therapy for bacterial meningitis as described elsewhere [7].

### Cause of death

Patient files were reviewed by expert infectious disease specialists in teams of two (CTB, JB, BRH and HN) whom in the daily clinical work treat patients with central nervous system infections. Autopsy results were available in eight cases. Deaths were categorized to be caused by either:

1. Central nervous system (CNS) complications
2. Systemic complications
3. Combination of systemic and CNS complications
4. Sudden death
5. Withdrawal of care
6. Cause of death could not be ascertained.

After each primary category had been determined, a specific diagnosis on the cause of death was assigned. In case of disagreement on the primary cause of death, a third clinical expert reviewed the case and a final diagnosis was established by discussion.

### Central nervous system (CNS) complications

#### Brain herniation

A diagnosis of brain herniation was applied to patients where a decline in consciousness was observed over hours combined with pupillary abnormalities and/or secondary circulatory or respiratory complications.

#### Brain injury

Brain infarct or brain hemorrhage was only ascertained as primary cause of death where a terminal event coincided with findings of large brain infarcts or brain hemorrhage without other apparent reasons for fatal outcome.

#### Intractable seizures

This diagnosis was applied to patients in whom maximum anticonvulsive therapy including respirator treatment was unable to stop seizure activity or general twitching.

#### Global injury / unresponsive

This diagnosis was assigned to patients with brain injury on imaging or with diminished pupillary reaction who did not receive sedatives.

### Systemic complications

*Circulatory failure* was determined as cause of death in cases with hypotension unresponsive to fluid and vasopressor therapy including patients where fatal circulatory failure led to cardiac arrest. This category represented cases with a clinical presentation resembling septic shock.

*Respiratory failure* was applied as cause of death in patients with respiratory fatigue, oxygenation problems also including patient with respiratory failure despite mechanical respirator treatment.

*Other organ failure* refers to death related to liver-failure, kidney-failure or intestinal complications.

### Other specified causes of death

*Sudden unexpected death* was defined by patients considered to be in a disease recovery phase and expected to recover for discharge by the treating clinician. Sudden death due to cardiac arrest in the acute phase of the disease was as primary group assigned to systemic complication and sub-categorized as circulatory failure.

*Withdrawal of care* was the cause of death in cases were suspected CNS complications, systemic complications or the combination where left untreated or patients taken off mechanical ventilator assistance due to other severe concurrent conditions and comorbidity.

### Statistical analysis

Categorical data are presented as absolute counts and percentages and, when relevant, compared by Fisher’s exact test or Chi-square. Continuous data are presented as median and inter-quartile range (IQR) and compared using the Kruskal-Wallis test. A Cohens *kappa* was calculated for the level of agreement between all raters for the primary and secondary causes of death. Correlation coefficients was assessed by the following modified Cohens scale; 0–0.20 = No agreement; 0.21 to 0.39 = Minimal agreement; 0.40 to 0.59 = Weak agreement; 0.60 to 0.79 = Moderate agreement; 0.80 to 0.9 = Strong agreement; Above 0.9 = Almost perfect agreement [11]. All calculations were carried out using PRISM 8.

## Results

A total of 358 patients with microbiologically confirmed bacterial meningitis were included in this study. Gender and age were equally distributed in the population with 179 males (50%) with a median age of 59 (48 to 68) years and 179 females with a median age of 59 years (50 to 72). Eighty-four patients (23%) died in-hospital. Median time to death was 8 days (3 to 16.8).

Mortality was unchanged in the study period (1998 to 2007: 22.2% and 2007 to 2015: 24.5%).

The no. of patients treated with adjunctive dexamethasone was not different between survivors and fatal cases (*n* = 146 of 274 versus *n* = 35 of 84, *p* = 0.08).

### Cause and time of death, Table 1 and Fig. 1

A primary cause of death could be ascertained in 82 of 84 patients (98%). Thirty-six patients (43%) died from CNS complications and 33 patients (39%) died from systemic complications. Fifteen patients (18%) were not classified into these two categories. Of these; Six patients (7%) died suddenly and unexpected; Care and treatment was withdrawn in 4 patients (5%); The cause of death was assigned to a combination of CNS and systemic causes in three patients (4%).

**Table 1:** The cause and time of death. Categorization of the causes of death into three admission-time categories - early, intermediate and late death. Specific causes of death are shown below the primary categories

Cause and time of death among 84 patients	Total n	Early time of death 0 to 2 days from admission	Intermediate time of death 3 to 14 days from admission	Late time of death > 14 days from admission
	**84**	**18 of 84** (21%)	**38 of 84** (45%)	**28 of 84** (33%)
**1. CNS complications**	**36 of 84** (43%)	8 of 18 (44%)	19 of 38 (50%)	9 of 28 (32%)
Brain herniation	**8**	6	2	0
Brain infarct	**6**	0	3	3
Brain haemorrhage	**1**	0	0	1
Intractable seizures	**6**	0	6	0
Global brain injury /unresponsive	**11**	0	6	5
CNS complications not further specified	**4**	2	2	0
**2. Systemic complications**	**33 of 84** (39%)	9 of 18 (50%)	13 of 38 (34%)	11 of 28 (39%)
Circulatory failure /septic shock	**14**	7	4	3
Respiratory failure	**7**	0	2	5
Other organ failure	**6**	1	4	1
Systemic complications not futher specified	**6**	1	3	2
**3. Combined CNS and systemic causes**	**3 of 84** (4%)	1 of 18 (6%)	0 of 38 (0%)	2 of 28 (7%)
**4. Sudden unexpected death**	**6 of 84** (7%)	0 of 18 (0%)	3 of 38 (8%)	3 of 28 (11%)
**5. Withdrawel of care**	**4 of 84** (5%)	0 of 18 (0%)	2 of 38 (5%)	2 of 28 (7%)
**6. Unable to determine cause of death**	**2 of 84** (2%)	0 of 18 (0%)	1 of 38 (3%)	1 of 28 (4%)

**Fig. 1:**
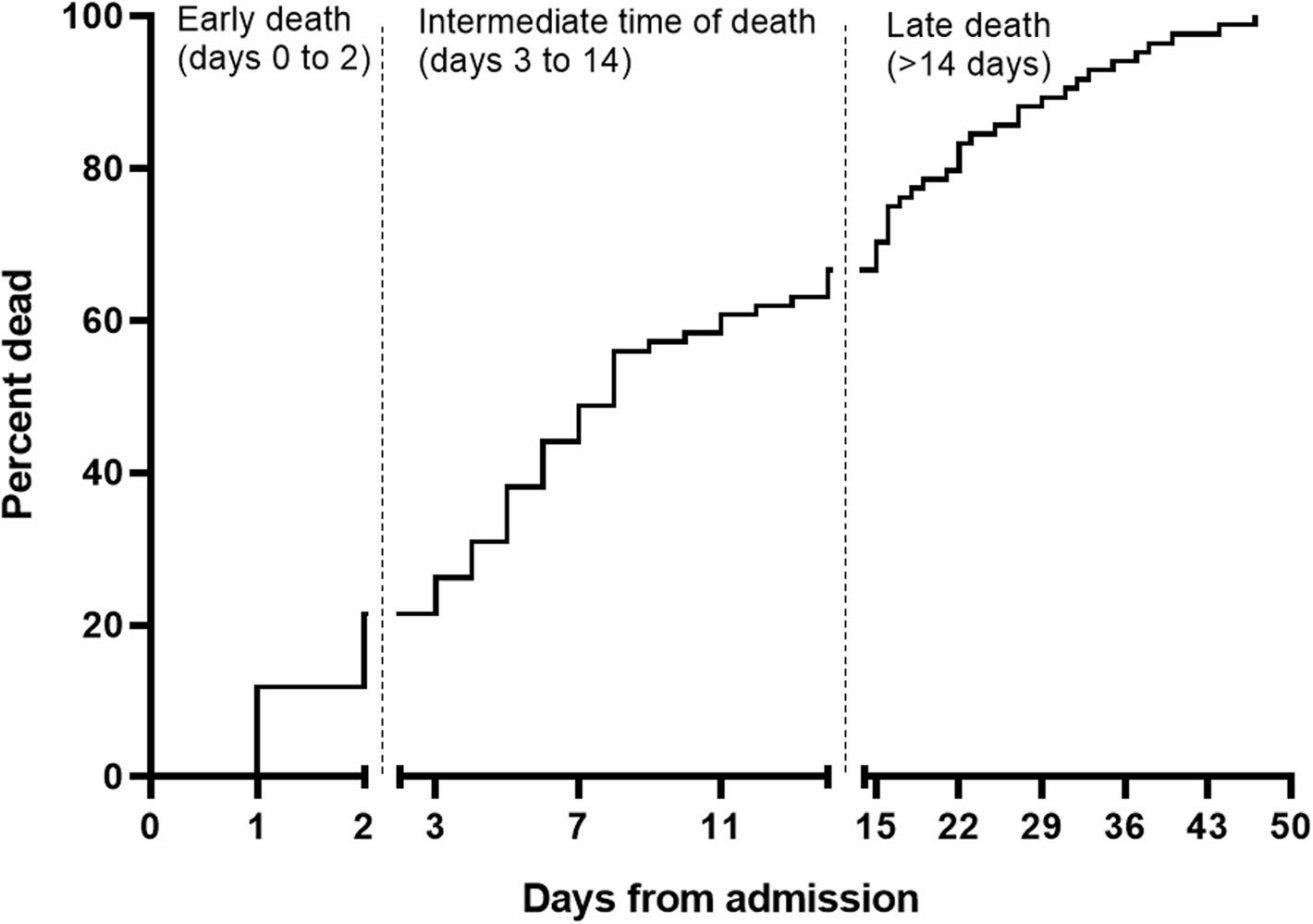
Time from admission to death. At 7 days from admission 41 of 84 patients were dead (49%). Within 14 days of admission 56 patients (66%) had died. Median time to death was 8 days (3 to 16.8)

#### Early mortality (< 48 h since admission)

Eighteen patients of 84 (21%) died soon after admission with causes equally distributed between CNS and systemic complications (8 and 9 patients respectively). One patient was classified with combined CNS and systemic complications as the cause of death (Table 1).

Brain herniation and circulatory failure were the specific causes of death in 13 of 18 (72%) patients dying within 2 days of admission.

#### Intermediate mortality (3 to 14 days after admission)

Thirty-eight of 84 patients (45%) died in this time interval. A CNS complication was the primary cause of death in 19 patients (50%) and systemic complications in 13 (34%). Sudden unexpected death, withdrawal of care and undetermined cause of death was observed in six cases (16%) (Table 1).

#### Late mortality (> 14 days after admission)

Twenty-eight of 84 patients (33%) died late during admission. CNS complications, systemic complications and the combined outcome of CNS and systemic complications was responsible for fatal outcome in eight (29%), 12 (43%) and two (7%) patients, respectively. Sudden unexpected death occurred in three patients (11%) and care was withdrawn in two patients (7%).

Brain herniation, global brain injury, intractable seizures and circulatory failure was determined cause of death in 31 of 56 patients (55%) dying within 14 days, Table 1.

### Interrater agreement

The level of agreement between all raters was substantial with a kappa 0.61 for primary classification of cause of death and 0.53 for the secondary subclassification.

### Fatal CNS and systemic complications – patient comparisons

#### Fatal CNS complications, Table 2

Clinically, patients with fatal outcome due to a CNS complication were older than survivors. The proportion of patients with a Glasgow Coma Score (GCS) below 9 as well as the proportion of patients with focal neurological deficits were higher among patients with fatal CNS complications (17 of 36 (47%) vs. 47 of 274 (18%), *p* < 0.0001) and (11 of 36 (21%) vs. 25 of 274 (9%), *p* = 0.0006) compared to survivors. Heart rate/pulse was significantly higher on admission compared to survivors (106 (88 to 120) vs. 95 (84 to 110), *p* = 0.022). Blood and CSF biochemistry was not different comparing patients dying from CNS complications to survivors.

**Table 2:** Baseline characteristics of patient with bacterial meningitis. Demographics-, clinical-, biochemical-, brain pathological- and causative pathogen data in 274 survivors from bacterial meningitis and 84 patients dying from bacterial meningitis

	Survivors	Died	Died from systemic complications	Died from CNS complications	*p*-value
	*n* = 274	*n* = 84	*n* = 33	*n* = 36	
Age (years)	56 (44 to 66)	69 (57 to 78)	67 (52 to 76)^a^	69 (54 to 77)^a^	0.0002
Male sex	145 (53%)	33 (40%)	13 (39%)	15 (42%)	0.08
Admission days	16 (12 to 28)	8 (3 to 16.8)	9 (2 to 16)	6 (3 to 14.8)	*ND*
**Clinical findings**					
Seizures before or on admission	28 (10%)	5 (6%)	1 (3%)	2 (6%)	0.33
Glasgow Coma Scale score < 9 (GCS)	47 (18%)	30 (37%)	6 (18%)	17 (47%)^a^	< 0.0001
Focal neurological deficits on admission	25 (9%)	19 (23%)	6 (18%)	11 (31%)^a^	0.0006
Focus of infection (otitis media/sinuitis)	73 (27%)	14 (17%)	2 (6%)^a^	7 (19%)	0.026
Focus of infection (pneumonia)	34 (12%)	15 (18%)	4 (12%)	7 (19%)	0.49
Systolic blood pressure on admission (mmHg)	138 (122 to 158)	135 (110 to 161)	120 (90 to 144)^a^	142 (121 to 180)	0.0023
Pulse rate (beats/minute)	95 (84 to 110)	106 (90 to 128)	105 (92 to 130)^a^	106 (88 to 120)^a^	0.0022
**Laboratory results**					
B-leucocytes (10^9^/L), *n* = 332	17.6 (12.6 to 23.9)	15 (9.9 to 20)	14.2 (9.2 to 19.0)^a^	15.0 (11.0 to 25.0)	0.035
B-thrombocytes (10^9^/L), *n* = 307	208 (145 to 273)	149 (73 to 244)	103 (57 to 203)^a^	206 (111 to 311)	< 0.0001
B-creatinine (mmol/L), *n* = 318	78 (63 to 100)	100 (77 to 190)	162 (76 to 227)^a^	91 (68 to 118)	0.0003
Cerebrospinal fluid-leucocytes (10^6^/L), *n* = 346	2653 (660 to 6152)	947 (165 to 5754)	600 (164 to 4976)	1040 (160 to 6229)	0.052
Cerebrospinal fluid-protein (g/L), *n* = 323	3.6 (1.9 to 6.5)	5.2 (1.4 to 8.7)	2.6 (0.7 to 7.6)	5.9 (2.4 to 8.8)	0.13
**Brain imaging pathology**					
No. of patients with brain imaging pathology	62 (23%)	35 (42%)	9 (27%)	23 (64%)^a^	< 0.0001
Brain infarction or haemorrhage	24 (9%)	21 (24%)	7 (21%)	14 (39%)^a^	< 0.0001
Brain hydrocephalus	5 (2%)	5 (6%)	0 (0%)	5 (14%)^a^	0.0002
Brain edema	15 (5.5%)	7 (8%)	1 (3%)	5 (14%)	0.10
**Microbiology**					
Patients with positive blood culture	150 (55%)	54 (64%)	23 (70%)	23 (66%)	0.18
*Streptococcus pneumoniae*	149 (54%)	45 (54%)	9 (27%)	25 (69%)	0.11
*Neisseria meningittidis*	54 (20%)	5 (6%)	5 (15%)	0 (0%)^a^	0.0009
*Staphylococcus aureus*	8 (3%)	13 (15%)	8 (24%)^a^	5 (14%)	< 0.0001
*Listeria monocytogenes*	18 (7%)	8 (10%)	4 (12%)	2 (6%)	0.47
Other streptococci	18 (7%)	8 (10%)	6 (18%)^a^	1 (3%)	0.27
Other gram negative bacteria	21 (8%)	5 (6%)	1 (3%)	3 (8%)	0.63

^a^Kruskal-Wallis test (medians with quartiles) or Fishers exact test, signicantly different comparing fatal outcome due to systemic and CNS complications to the group of survivors

*ND* Not Determined

Brain pathology visualized on brain imaging was significantly more common among patients dying from CNS complications than among survivors (23 of 36 (64%) vs. 62 of 274 (23%), *p* < 0.0001).

Microbiology*. S. pneumoniae* was the most common causative organism in patient dying from CNS complications but not different to survivors (25 of 36 (69%) vs. 149 of 274 (54%), *p* = 0.11). No cases of meningitis due to *N. meningitidis* died from CNS complications.

#### Fatal systemic complications, Table 2

Clinically, the heart rate/pulse was significantly higher on admission in patients dying from systemic complications compared to survivors (105 (92 to 130) vs. 95 (84 to 110), *p* = 0.0022).

Otitis media or sinusitis as focus of infection was significantly less common among patients dying from systemic complications compared to survivors (2 of 33 (6%) vs. 73 of 274 (27%), *p* = 0.026).

Blood and CSF biochemistry data showed significantly decreased levels of blood leukocytes and thrombocytes among patients dying from systemic complications compared to survivors (leukocyte count 14.2 × 10^9^ cells/L (9.2 to 19.0) vs. 17.6 (12.6 to 23.9), *p* = 0.035), and thrombocyte count 103 × 10^9^ cells/L (57 to 203) vs. 208 (145 to 273), *p* < 0.0001). Blood creatinine levels were significantly increased in patients dying from systemic complication compared to survivors (162 mmol/L (76 to 227) vs. 78 (62 to 100), *p* = 0.0003). Cerebrospinal fluid (CSF) leukocyte count was borderline significantly lower among patients dying from systemic complications compared to survivors (600 × 10^6^ cells/L (164 to 4976) vs. 2653 (660 to 6152), *p* = 0.052).

Brain pathology visualized on brain imaging was not different comparing patients dying from systemic complications to survivors.

Microbiology*.* Among patients dying from systemic complications, infection due to *S. aureus* was more common than among survivors (8 of 33 (24%) vs. 8 of 274 (3%), *p* < 0.0001).

Clinical-, biochemical-, brain imaging-, and microbiological data for the most common specific causes of death within 14 days of admission are shown in Table 3.

**Table 3:** Baseline demographics-, clinical-, biochemical-, brain pathological- and causative pathogen data for the 4 most prevalent specific causes of death occurring within 14 days from admission

Baseline characteristics of patients dying from bacterial meningitis	Brain herniation	Global brain injury /unresponsive	Intractable seizures	Circulatory failure
	*n* = 8	*n* = 11	*n* = 6	*n* = 14
Age (years)	52 (38 to 75)	68 (54 to 74)	70 (62 to 83)	55 (47 to 69)
Male sex	2 (25%)	4 (36%)	4 (67%)	4 (29%)
Survival days	2 (1 to 2.8)	10 (6 to 18)	4.5 (3.8 to 5.8)	3 (1.8 to 16.8)
**Clinical findings**				
Seizures before or on admission	0 (0%)	0 (0%)	1 (17%)	1 (7%)
Glasgow Coma Scale score < 9 (GCS)	4 (50%)	7 (64%)	2 (34%)	3 (21%)
Focal neurological deficits on admission	2 (25%)	3 (27%)	1 (17%)	4 (29%)
Focus of infection (otitis media/sinuitis)	3 (38%)	2 (18%)	0 (0%)	0 (0%)
Focus of infection (pneumonia)	2 (25%)	2 (18%)	2 (33%)	2 (14%)
Systolic blood pressure on admission (mmHg)	145 (120 to 181)	172 (120 to 210)	160 (122 to 170)	106 (84.5 to 122.8)
Pulse rate (beats/minute)	83 (78 to 128)	110 (103 to 129)	102 (86 to 120)	130 (93 to 186)
**Laboratory results**				
B-leucocytes (10^9^/L)	18.3 (8.9 to 24.5)	16.5 (12.8 to 26.9)	11.6 (7.7 to 25.0)	15.7 (6.4 to 20.5)
B-thrombocytes (10^9^/L)	183 (101 to 286)	304 (128 to 452)	208 (167 to 238)	99 (68 to 201)
B-creatinine (mmol/L)	75 (63 to 90)	69 (52 to 102)	112 (95 to 141)	197 (95 to 247)
Cerebrospinal fluid-leucocytes (10^6^/L)	2742 (474 to 54.800)	1040 (741 to 4330)	932 (115 to 5326)	558 (75 to 4566)
Cerebrospinal fluid-protein (g/L)	4.6 (2.8 to 9.9)	6.1 (2.1 to 9.1)	5.5 (3.6 to 7.2)	1.9 (0.6 to 15.9)
**Brain imaging pathology**				
No. of patient with brain imaging pathology	7 (88%)	8 (72%)	0 (0%)	3 (23%)
Brain infarction or haemorrhage	1 (13%)	6 (55%)	0 (0%)	2 (15%)
Brain Hydrocephalus	2 (25%)	3 (27%)	0 (0%)	0 (0%)
Brain edema	5 (63%)*	0 (0%)	0 (0%)	0 (0%)
**Microbiology**				
Patients with positive blood culture	5 (63%)	6 (55%)	4 (67%)	11 (79%)
Streptococcus pneumoniae	6 (75%)	7 (64%)	5 (83%)	4 (29%)
Neisseria meningittidis	0 (0%)	0 (0%)	0 (0%)	3 (21%)
Staphylococcus aureus	0 (0%)	2 (18%)	0 (0%)	4 (29%)
Listeria	0 (0%)	1 (9%)	1 (17%)	0 (0%)
Other streptococci	0 (0%)	1 (9%)	0 (0%)	3 (21%)
Other gram negative bacteria	2 (25%)	0 (0%)	0 (0%)	0 (0%)

The cause of death according to GCS on admission are shown in Table 4.

**Table 4:** Cause of death and GCS level on admission. Data was available for 82 of 84 patients. The no. of patients presenting with a GCS < 9 was significantly higher among patients dying from CNS complications than among patients dying from systemic complications (*p* = 0.011)

	GCS < 9	GCS 9–12	GCS 13–14	GCS 15
Data available for 82 of 84 patients	**30 of 82** (37%)	**29 of 82** (35%)	**16 of 82** (20%)	**7 of 82** (9%)
**1. CNS complications**	17 of 30 (57%)	9 of 29 (31%)	7 of 16 (44%)	2 of 7 (29%)
Brain herniation	4	3	0	1
Brain infarct	1	3	1	0
Brain haemorrhage	1	0	0	0
Intractable seizures	2	1	2	1
Global brain injury /unresponsive	7	0	4	0
CNS complications not further specified	2	2	0	0
**2. Systemic complications**	6 of 30 (20%)	15 of 29 (52%)	8 of 16 (50%)	4 of 7 (57%)
Circulatory failure /septic shock	2	6	2	3
Respiratory failure	0	5	2	0
Other organ failure	0	1	2	0
Systemic complications not futher specified	4	3	2	1
**3. Combined CNS and systemic causes**	2 of 30 (7%)	0 of 39 (0%)	1 of 16 (6%)	0 of 7 (0%)
**4. Sudden unexpected death**	3 of 30 (10%)	2 of 29 (7%)	0 of 16 (0%)	0 of 7 (0%)
**5. Withdrawel of care**	2 of 30 (7%)	1 of 29 (7%)	0 of 16 (0%)	1 of 7 (14%)
**6. Unable to determine cause of death**	0 of 30 (0%)	2 of 29 (7%)	0 of 16 (0%)	0 of 7 (0%)

The proportion of patients presenting with unconsciousness (GCS < 9) was higher among patient dying from CNS complications compared to patients dying from systemic complications (17 of 30 (57%) versus 6 of 30 (20%), *p* = 0.011). In total only 7 patients dying from meningitis presented with GCS 15 (9%).

Timing of antibiotic therapy was subject to an equal delay among patient dying from CNS or systemic complications (median 4.4 versus 5.3 h, *p* = 0.65), Table 5. Among patients dying from CNS complications patients dying from brain herniation was treated most urgently whereas patients dying from brain infarction was subject to the greatest treatment delay (ns).

**Table 5:** Cause of death and timing of appropriate antibiotic treatment for bacterial meningitis. Data was available for 74 of 84 patients. No significant differences in time to treatment for bacterial meningitis was found (Kruskal-Wallis test, *p* > 0.05)

	Time to treatment for bacterial meningitis	0 to 2 h	2 to 6 h	> 6 h
Data available for 74 of 84 patients	Hours (median and quartiles)	**23 of 74** (31%)	**16 of 74** (22%)	**35 of 74** (47%)
**1. CNS complications (*n* = 32)**	4.4 (1.3 to 22.4)	11 of 23 (48%)	6 of 16 (38%)	15 of 35 (43%)
Brain herniation (*n* = 7)	1.0 (0.5 to 8.6)	5	0	2
Brain infarct (*n* = 5)	25.0 (5.2 to 52.5)	1	0	4
Brain haemorrhage (*n* = 1)	2.0	1	0	0
Intractable seizures (*n* = 6)	11.0 (2.3 to 31.0)	1	2	3
Global brain injury /unresponsive (*n* = 9)	10.5 (3.3 to 34.8)	1	2	6
CNS complications not further specified (*n* = 4)	2.4 (1.3 to 4.5)	2	2	0
**2. Systemic complications (*n* = 28)**	5.3 (1.9 to 21.5)	7 of 23 (30%)	8 of 16 (50%)	13 of 35 (37%)
Circulatory failure /septic shock (*n* = 10)	8.2 (1.0 to 40.8)	3	1	6
Respiratory failure (*n* = 7)	6.0 (3.5 to 20.0)	1	3	3
Other organ failure (*n* = 2)	9.5 (1.0 to 18.0)	1	0	1
Systemic complications not futher specified (*n* = 9)	3.9 (1.9 to 18.2)	2	4	3
**3. Combined CNS and systemic causes (*n* = 3)**	1.3 (0.6 to 4.5)	2 of 23 (9%)	1 of 16 (6%)	0 of 35 (0%)
**4. Sudden unexpected death (*n* = 5)**	18.0 (1.9 to 39.0)	1 of 23 (4%)	1 of 16 (6%)	3 of 35 (9%)
**5. Withdrawel of care (*n* = 4)**	4.2 (0.9 to 20.0)	2 of 23 (9%)	0 of 16 (0%)	2 of 35 (6%)
**6. Unable to determine cause of death (*n* = 2)**	25.1 (24.0 to 26.3)	0 of 23 (0%)	0 of 16 (0%)	2 of 35 (6%)

## Discussion

In the present study, we evaluated the causes and timely occurrence of death in bacterial meningitis in adults. We identified a wide spectrum of complications ranging from brain edema and vascular injury to circulatory- and respiratory failure. Our findings corroborate that assigning death only to meningitis is a crude measure.

We were able to categorize the cause of death in 82 of 84 cases. Sixty-nine of 82 cases were categorized into two main causes of death - CNS and systemic complications.

Among patients dying within 48 h of admission, 13 of 18 patients (72%) died from two distinct acute meningitis complications – brain herniation or circulatory failure due to septic shock.

Meningitis patients categorized as dying from systemic complications presented with a clinical appearance, blood biochemistry, microbiological findings and brain pathology different to survivors and patients dying from CNS complications. Also, the proportion of patients presenting with low GCS on admission was high among patient dying from CNS complications whereas patient dying from systemic complications less commonly presented with unconsciousness (Table 4). Despite the very different clinical and paraclinical presentations, patient dying from CNS complications did not receive more prompt treatment for bacterial meningitis except for those with early fatal disease caused by brain herniation. These findings, that essentially depict the characteristics of septicemia versus classical meningitis pathology, may not be a surprising result.

The very different courses of disease that lead to death from meningitis emphasize the need for a nuanced view on fatal cases. Categorizing the cause of death as well as characterizing each case has also been suggested in studies of the cause of death from sepsis [12–14]. The goal is to be able to prevent complications and to improve the validity of clinical trials by improving study homogeneity.

Two previous studies by Weisfelt et al. [2, 3] investigated the cause of death from bacterial meningitis and more specifically pneumococcal meningitis. Our results concerning categorization of the cause of death and distribution between systemic and CNS complications causing of death are very similar. Also, Weisfelt et al. showed that brain herniation is primarily observed among younger patients which we believe agrees with our findings (Table 3). Only did Weisfelt et al. find that more patients with pneumococci died from systemic causes where pneumococci in our study was the primary cause of CNS complications.

CNS complications represented 43% of the total causes of death. Most fatal cases due to CNS complications (75%) was observed within 14 days of admission. This is in agreement with the results by McMillan et al. [5]*,* who found that death due to the meningeal infection itself was less likely to occur more than 14 days after admission. After 14 days, systemic complications that were more likely due to secondary infections, were also more common in our cohort (Table 1). The timely distribution of death in our population was almost identical to the findings in the study by McMillan et al. (Fig. 1).

Previously identified prognostic factors for poor outcome from meningitis in adults are advanced age, low GCS on admission, positive blood culture, low CSF leukocyte count, elevated CSF protein and immunosuppressive comorbidity including alcoholism [15–17]. Also, neurological deficits and presence of seizures on admission are indicators of a poor prognosis [18]. These parameters cannot be translated into treatment strategies improving the course of disease albeit knowledge of increased risk of meningitis among certain groups could led to early empiric treatment or prevention with vaccines [7]. Even though speculative, the clinical, biochemical and brain imaging description of patients dying from bacterial meningitis could be very important for the discovery of modifiable factors that could improve outcome due to early identification of patients at risk. Similar considerations have previously been published in the field of septicemia as referenced above.

The present study has obvious limitations due to the retrospective data-collection. The presented data are dependent on the attending physician’s description of the disease and its complications. Even though patients were admitted to departments specialized in the treatment and care for patients with bacterial meningitis the level of detail in the medical records varied. The few autopsies performed did only support the clinical diagnosis and previous autopsy studies in patients dying from sepsis or meningitis have not identified the cause of death but have provided vital information of the organ injury [19, 20]. In that respect we believe that the cause of death analysis performed by clinical experts must be second best to the combination of autopsy and clinical case evaluation.

In a rather large proportion of our patients (21%) we were unable to determine a more specific diagnosis as the cause of death which may pose a significant problem to the timely assessment of cause of death. Among the group of patients where we were unable to provide a more specific diagnosis than CNS complications and the group suggested to have global brain injury/unresponsive state may have suffered an unregistered critical event including subtle signs of brain herniation/edema or a period with circulatory insufficiency. Also, the rater could be biased towards a diagnosis of a CNS complication in patients with an unresponsive state after several days or weeks in hospital care. Eventually, there is a risk that these patients were wrongly labelled due to the retrospective clinical evaluation.

Our interrater agreement is comparable to other previous studies and we find the agreement to be acceptable [2, 14]. The review of cases with disagreement with a third expert resulted in full agreement.

## Conclusion

The cause of death from meningitis is very diverse but can on a superior level be divided into local CNS- or systemic complications. CNS complications present with abnormal brain imaging in 2/3 of cases and are most commonly caused by pneumococci. Systemic complications are primarily circulatory failure presenting with clinical and biochemical sepsis. Death related to the primary infection – meningitis – occur most commonly within 14 days from admission.

Determining the cause of death from meningitis should be a priority in the evaluation of clinical trials.

## Data Availability

According to Danish law, permissions from the Danish Data Protection Agency and the Danish Board of Health are required before patient data can be shared by request from a qualified researcher. Data are available upon request to the corresponding author (CTB).

## Abbreviations

CNS: Central nervous system
CSF: Cerebrospinal fluid
GCS: Glasgow Coma Score
IQR: Interquartile Range
